# Validation List no. 218. Valid publication of new names and new combinations effectively published outside the IJSEM

**DOI:** 10.1099/ijsem.0.006398

**Published:** 2024-07-31

**Authors:** Aharon Oren, Markus Göker

**Affiliations:** 1 The Institute of Life Sciences, The Hebrew University of Jerusalem, The Edmond J. Safra Campus, 9190401 Jerusalem, Israel; 2 Leibniz Institute DSMZ – German Collection of Microorganisms and Cell Cultures, Inhoffenstrasse 7B, 38124 Braunschweig, Germany

The purpose of this list is to publish in the *International Journal of Systematic and Evolutionary Microbiology* (IJSEM) those new names and new combinations that were effectively published outside the IJSEM as set forth in Rule 27 of the International Code of Nomenclature of Prokaryotes (2022 Revision) [1]. Authors and other individuals wishing to have new names and/or combinations included in future lists should send an electronic copy of the published paper to the IJSEM Editorial Office for confirmation that all of the other requirements for valid publication have been met. It is also a requirement of IJSEM and the International Code of Nomenclature of Prokaryotes (ICNP) that authors of new species, new subspecies and new combinations provide evidence that types are deposited in two recognized culture collections in two different countries. It should be noted that the date of valid publication of these new names and combinations is the date of publication of this list, not the date of the original publication of the names and combinations. The authors of the new names and combinations are as given below.

Inclusion of a name on these lists validates the publication of the name and thereby makes it available in the nomenclature of prokaryotes. Inclusion of a name on these lists is part of the requirements of valid publication of an effectively published name. Indeed, some of these names may, in time, be considered later synonyms, or it may be proposed to transfer the organisms to another genus, thus necessitating the creation of a new combination. Conversely, it may be proposed to transfer an organism back to an earlier genus and to regard the basonym or an earlier new combination as the correct name instead of a more recent new combination. The ICNP does not decide on such taxonomic opinions.

**Table:** 

Name/authors	Proposed as	Nomenclatural type^1^	Priority^2^	Reference
*Actinomycetospora lemnae* Saimee *et al*. 2024, 11^3^	sp. nov.	Dw7h6 (=NBRC 115294=TBRC 15165)	15	[2]
*Aedoeadaptatus coxii* (Citron *et al*. 2013) Malhotra *et al*. 2024, 15^3^	comb. nov. [basonym: *Peptoniphilus coxii* Citron *et al*. 2013]^4^	RMA 16757 (=ATCC BAA-2106=CCUG 59622=JCM 16892)	21	[3]
*Aedoeadaptatus ivorii* (Murdoch *et al*. 1997) Malhotra *et al*. 2024, 16^3^	comb. nov. [basonym: *Peptostreptococcus ivorii* Murdoch *et al*. 1997]^4^	CCUG 38492 (=DSM 10022=NCTC 13078)^5^	21	[3]
*Aedoeadaptatus nemausensis* (Aujoulat *et al*. 2022) Malhotra *et al*. 2024, 16^3^	comb. nov. [basonym: *Peptoniphilus nemausensis* Aujoulat *et al*. 2022]	1804121828 (=CECT 9935=LMG 31466)	21	[3]
*Aequorivita aurantiaca* Liu *et al*. 2023, 10^3^	sp. nov.	SDUM287046 (=KCTC 92754=MCCC 1H01418)	37	[4]
*Aequorivita marisscotiae* Liu *et al*. 2023, 2^3^	sp. nov.	Ant34-E75 (=CCTCC AB 2023079=KCTC 92993)	50	[5]
*Aestuariimicrobium ganziense* Geng *et al*. 2021, 2655	sp. nov.	YIM S02566 (=CGMCC 1.18751=KCTC 49477)^6^	43	[6]
*Amycolatopsis solani* Wannawong *et al*. 2024, 17^3^	sp. nov.	MEP2-6 (=JCM 36309=NBRC 116395)^7^	51	[7]
*Ancylobacter mangrovi* Li *et al*. 2023, 7^3^	sp. nov.	GSK1Z-4-2 (=JCM 34924=MCCC 1K07181)	11	[8]
*Aquitalea aquatica* De León *et al*. 2024, 1^3^	sp. nov.	HSC-21Su07 (=DSM 114499=NRRL B-65646)	6	[9]
*Brevibacillus daliensis* Ye *et al*. 2023, 4^3,8^	sp. nov.	YIM B02290 (=CCTCC AB 2021094=CGMCC 1.18802=KCTC 43376)	40	[10]
*Chlamydia caviae* (Everett *et al*. 1999) Sachse *et al*. 2015, 102	comb. nov. [basonym: *Chlamydophila caviae* Everett *et al*. 1999]^4,9^	GPIC (=ATCC VR813=DSM 19441)	46	[11]
*Chromobacterium fluminis* De León *et al*. 2024, 1^3^	sp. nov.	HSC-31F16 (=DSM 114492=NRRL B-65644)	6	[9]
*Clostridium lamae* Li *et al*. 2024, 8^3^	sp. nov.	NGMCC 1.200840 (=CGMCC 1.18014=JCM 35704)	9	[12]
*Cohnella mopanensis* Tang *et al*. 2022, 4^3^	sp. nov.	YIM B01951 (=KCTC 43370=NBRC 115331)	42	[13]
*Collinsella acetigenes* Han *et al*. 2021, 3671	sp. nov.	KGMB02528 (=CCUG 73987=KCTC 15847)	34	[14]
*Corallococcus soli* Babadi *et al*. 2022, 14^3,10^	sp. nov.	ZKHCc1 1396 (=CIP 111634=NCCB 100659)	16	[15]
*Demequina lignilytica* Gao *et al*. 2023, 11^3^	sp. nov.	SYSU T00068 (=GDMCC 1.3838=KCTC 49954)	39	[16]
*Demequina litoralis* Gao *et al*. 2023, 11^3^	sp. nov.	SYSU T00192 (=GDMCC 1.3840=KCTC 49956)	39	[16]
*Demequina muriae* Gao *et al*. 2023, 11^3^	sp. nov.	EGI L300058 (=GDMCC 1.3270=KCTC 59052)	39	[16]
*Demequina zhanjiangensis* Gao *et al*. 2023, 11^3^	sp. nov.	SYSU T00b26 (=GDMCC 1.3841=KCTC 49950)	39	[16]
*Devosia lacusdianchii* Deng *et al*. 2024, 12^3^	sp. nov.	JXJ CY 41 (=CGMCC 1.17502=KCTC 72812)	22	[17]
*Duganella violaceipulchra* De León *et al*. 2024, 2^3^	sp. nov.	HSC-15S17 (=ATCC TSD-229=CCOS 2056)	6	[9]
*Ensifer aridi* Rocha *et al*. 2020, 320^11^	sp. nov.	LMR001 (=HAMBI 3707=LMG 31426)	26	[18]
*Ensifer oleiphilus* Ershov *et al*. 2023, 15^3^	sp. nov.	HO-A22 (=KCTC 92427=VKM B-3646)	1	[19]
*Flavobacterium panacagri* Kim *et al*. 2023, 897	sp. nov.	CJ75 (=JCM 36132=KACC 23149)	10	[20]
*Flavobacterium psychrotrophum* Kim *et al*. 2023, 897	sp. nov.	CJ74 (=JCM 32889=KACC 19819)	10	[20]
*Fluctibacter* Emsley *et al*. 2024, 12^3^	gen. nov.	*Fluctibacter halophilus* ^12^	19	[21]
*Fluctibacter corallii* Emsley *et al*. 2024, 12^3^	sp. nov.	AA17 (=LMG 32603=NCTC 14664)	19	[21]
*Fluctibacter halophilus* (Yi *et al*. 2004) Emsley *et al*. 2024, 12^3^	comb. nov. [basonym: *Aestuariibacter halophilus* Yi *et al*. 2004]^13^	JC2043 (=DSM 15266=KCTC 12043)^14^	19	[21]
*Fundicoccus culcitae* Zhou *et al*. 2023, 1192	sp. nov.	DM20194951 (=GDMCC 1.3614=KCTC 43472)	48	[22]
*Gordonibacter faecis* Kim *et al*. 2024, 6^3^	sp. nov.	KGMB12511 (=KCTC 25343=NBRC 116190)	33	[23]
*Halobaculum halobium* Tan *et al*. 2024, 9^3^	sp. nov.	SYNS20 (=CGMCC 1.62628=JCM 36154)	47	[24]
*Halobaculum limi* Tan *et al*. 2024, 10^3^	sp. nov.	YSMS11 (=CGMCC 1.18927=JCM 34912)	47	[24]
*Halobaculum lipolyticum* Tan *et al*. 2024, 8^3^	sp. nov.	DT31 (=CGMCC 1.18923=JCM 35417)	47	[24]
*Halobaculum litoreum* Tan *et al*. 2024, 9^3^	sp. nov.	DT92 (=CGMCC 1.19057=JCM 36148)	47	[24]
*Halobaculum marinum* Tan *et al*. 2024, 9^3^	sp. nov.	DT55 (=CGMCC 1.19048=JCM 36147)	47	[24]
*Haloglomus litoreum* Hu *et al*. 2024, 8^3^	sp. nov.	DT116 (=CGMCC 1.19045=JCM 35606)	30	[25]
*Halomonas kalidii* Xu *et al*. 2024, 9^3^	sp. nov.	M4R5S39 (=CGMCC 1.62047=KCTC 8015)	20	[26]
*Halomonas maris* Qiu *et al*. 2021, 3284	sp. nov.	QX-1 (=DSM 112092=KCTC 82198=MCCC 1A17875)^15^	27	[27]
*Halomonas piscis* Kim *et al*. 2023, 8^3,16^	sp. nov.	SG2L-4 (=JCM 35929=KCTC 92842)	8	[28]
*Halomonas rhizosphaerae* Xu *et al*. 2024, 9^3^	sp. nov.	LR5S13 (=CGMCC 1.62049=KCTC 8016)	20	[26]
*Halomonas sedimenti* Qiu *et al*. 2021, 1667	sp. nov.	QX-2 (=DSM 112091=KCTC 82199=MCCC 1A17876)	28	[29]
*Halomontanus* Mao *et al*. 2024, 9^3^	gen. nov.	*Halomontanus rarus*	13	[30]
*Halomontanus rarus* Mao *et al*. 2024, 9^3^	sp. nov.	TS33 (=KCTC 4310=MCCC 4K00132)	13	[30]
*Halorarum* Tan *et al*. 2024, 8^3^	gen. nov.	*Halorarum halophilum*	47	[24]
*Halorarum halophilum* (Cui *et al*. 2021) Tan *et al*. 2024, 8^3^	comb. nov. [basonym: *Halobaculum halophilum* Cui *et al*. 2021]	Gai3-2 (=CGMCC 1.16080=JCM 33550)	47	[24]
*Halorarum salinum* (Cui *et al*. 2021) Tan *et al*. 2024, 8^3^	comb. nov. [basonym: *Halobaculum salinum* Cui *et al*. 2021]	NJ-3-1 (=CGMCC 1.16040=JCM 33552)	47	[24]
*Halorussus caseinilyticus* Hu *et al*. 2024, 9^3^	sp. nov.	DT72 (=CGMCC 1.18925=JCM 35418)	30	[25]
*Halorussus lipolyticus* Hu *et al*. 2024, 9^3^	sp. nov.	DT80 (=CGMCC 1.18926=JCM 35419)	30	[25]
*Halosegnis marinus* Hu *et al*. 2024, 8^3^	sp. nov.	DT85 (=CGMCC 1.19049=JCM 35605)	30	[25]
*Iodobacter violaceini* De León *et al*. 2024, 2^3^	sp. nov.	HSC-16F04 (=DSM 114489=NRRL B-65647)	6	[9]
*Jatrophihabitans cynanchi* Suh *et al*. 2024, 6^3^	sp. nov.	SB3-54 (=KCTC 49134=NBRC 114108)	36	[31]
*Jeongeupella* corrig. Jiang *et al*. 2023, 13^3,17^	gen. nov.	*Jeongeupella avenae* corrig.	3	[32]
*Jeongeupella avenae* corrig. Jiang *et al*. 2023, 13^3^	sp. nov.	DY-R2A-6 (=GDMCC 1.3014=KCTC 82985)	3	[32]
*Legionella resiliens* Cristino *et al*. 2024, 20^3^	sp. nov.	8cVS16 (=CCUG 76627=DSM 114356)	38	[33]
*Marinobacter suaedae* corrig. Park *et al*. 2024, 10^3,18^	sp. nov.	chi1 (=KACC 23259=TBRC 17652)	18	[34]
*Massilia endophytica* Jeon *et al*. 2023, 8^3^	sp. nov.	DM-R-R2A-13 (=GDMCC 1.2920=KCTC 92072)^19^	4	[35]
*Massilia hydrophila* De León *et al*. 2024, 3^3^	sp. nov.	HSC-2F05 (=DSM 114498=NRRL B-65648)	6	[9]
*Micrococcus lacusdianchii* Wang *et al*. 2024, 167	sp. nov.	JXJ CY 30 (=CGMCC 1.17508=KCTC 49378)	23	[36]
*Mucilaginibacter lacusdianchii* Xiao *et al*. 2024, 14^3^	sp. nov.	JXJ CY 39 (=CGMCC 1.17449=KCTC 72617)	24	[37]
*Oceanoferula flava* corrig. Ye *et al*. 2023, 9^3,20^	sp. nov.	5K15 (=KCTC 82758=MCCC 1H00442)^21^	17	[38]
*Planctellipticum* corrig. Wurzbacher *et al*. 2024, 11^3,22^	gen. nov.	*Planctellipticum variicoloris* corrig.	31	[39]
*Planctellipticum variicoloris* corrig. Wurzbacher *et al*. 2024, 11^3^	sp. nov.	SH412 (=CECT 30430=JMRC STH00996	31	[39]
*Prodigiosinella* Hugouvieux-Cotte-Pattat *et al*. 2024, 12^3^	gen. nov.	*Prodigiosinella aquatilis*	25	[40]
*Prodigiosinella aquatilis* Hugouvieux-Cotte-Pattat *et al*. 2024, 12^3^	sp. nov.	LS101 (=CFBP 8826=LMG 32072)	25	[40]
*Pseudidiomarina terrestris* Galisteo *et al*. 2024, 16^3^	sp. nov.	1APP75-27a (=CECT 30242=CCM 9142)	7	[41]
*Pseudomonas abieticivorans* Ristinmaa *et al*. 2023, 11^3^	sp. nov.	PIA16 (=CCUG 76343=DSM 114633)	5	[42]
*Pseudomonas maioricensis* Mulet *et al*. 2024, 16^3,23^	sp. nov.	S25 (=CCUG 69272=CECT 30911)	12	[43]
*Pseudomonas subflava* Ling *et al*. 2023, 562	sp. nov.	YIM B01952 (=CCTCC AB 2021498=KCTC 92073)	45	[44]
*Pseudonocardia lacus* Liu *et al*. 2024, 7^3^	sp. nov.	H11425 (=CICC 25118=JCM 34851)	49	[45]
*Rhizosphaericola* Kim *et al*. 2024, 7^3^	gen. nov.	*Rhizosphaericola mali* ^24^	35	[46]
*Rhizosphaericola mali* Kim *et al*. 2024, 7^3^	sp. nov.	B3-10 (=KCTC 72123=NBRC 114178)	35	[46]
*Sciscionella sediminilitoris* Teo *et al*. 2024, 6^3^	sp. nov.	SE31 (=DSM 46824=TBRC 5134)	52	[47]
*Selenomonas ruminis* Poothong *et al*. 2024, 5^3^	sp. nov.	mPRGC5 (=JCM 33724=KCTC 25177)	14	[48]
*Solibacillus ferritrahens* Liu *et al*. 2024, 8^3^	sp. nov.	YIM B08730 (=CGMCC 1.60169=NBRC 116268)	44	[49]
*Thalassococcus arenae* Weerawongwiwat *et al*. 2022, 6^3^	sp. nov.	CAU 1522 (=KCTC 72545=MCCC 1K04064)	2	[50]
*Thiopseudomonas acetoxidans* An *et al*. 2024, 8^3^	sp. nov.	CY1220 (=GDMCC 1.3503=JCM 35747)	32	[51]
*Viridibacillus soli* Xu *et al*. 2022, 4^3^	sp. nov.	YIM B01967 (=CGMCC 1.18436=KCTC 43249)^25^	41	[52]
*Wenyingzhuangia gilva* Park *et al*. 2024, 10^3,26^	sp. nov.	chi5 (=KACC 23262=TBRC 17900)	18	[34]

For references to Validation Lists 1–71, see *Int J Syst Bacteriol* **49** (1999) 1325. Lists 72–197 were published in *Int J Syst Evol Microbiol* **50** (2000) 3, 423, 949, 1415, 1699, 1953; and **51** (2001) 1, 263, 793, 1229, 1619, 1945; and **52** (2002) 3, 685, 1075, 1437, 1915; and **53** (2003) 1, 373, 627, 935, 1219, 1701; and **54** (2004) 1, 307, 631, 1005, 1425, 1909; and **55** (2005) 1, 547, 983, 1395, 1743, 2235; and **56** (2006) 1, 499, 925, 1459, 2025, 2507; and **57** (2007) 1, 433, 893, 1371, 1933, 2449; and **58** (2008) 1, 529, 1057, 1511, 1993, 2471; and **59** (2009) 1, 451, 923, 1555, 2129, 2647; and **60** (2010) 1, 469, 1009, 1477, 1985, 2509; **61** (2011) 1, 475,1011, 1499, 2025, 2563; and **62** (2012) 1, 473, 1017, 1443, 2045, 2549; and **63** (2013) 1, 797, 1577, 2365, 3131, 3931; and **64** (2014) 1, 693, 1455, 2184, 3603; and **65** (2015), 1, 741, 1112, 2017, 2777, 3767; and **66** (2016) 1, 1603, 1913, 2463, 3761, 4299; and **67** (2017) 1, 529, 1095, 2075, 3140, 4291; and **68** (2018), 1, 693, 1411, 2130, 2707, 3379; and **69** (2019), 5, 597, 1247, 1844, 2627, 3313, and **70** (2020) 1, 1443, 2960, 4043, 4844, 5596. Lists 197–217 were published in *Int J Syst Evol Microbiol* **71** (2021) 004600, 004688, 004773, 004846, 004943, 005096; and **72** (2022) 005167, 005260, 005331, 005422, 005517, 005592; and **73** (2023) 005709, 005812, 005845, 005931, 005997, 006080; and **74** (2024) 006173, 006229, 006275.

1Abbreviations of culture collections cited in this list can be found at https://lpsn.dsmz.de/text/collections.https://lpsn.dsmz.de/text/collections

2Priority number assigned according to the date the documentation and request for validation are received.

3The journal in which the name was effective published does not have continuous pages.

4The basonym is classified as Risk Group 2.

5The effective publication states that the type strain was also deposited as CIP 105325, but no documentation was supplied.

6In the abstract, the KCTC number is erroneously given as KCTC 49,477.

7The effective publication states that the type strain was also deposited as TBRC 17632, but no documentation was supplied.

8The etymology should state N.L. masc. adj.

9The basonym was not explicitly mentioned in the article.

10The protologue heading should state sp. nov. instead of sp. Nov.

11The etymology should state a’ri.di. L. gen. n. *aridi*, of a dry place.

12The protologue erroneously states *F. halophilus* instead of *Fluctibacter halophilus*.

13The basonym was erroneously given as *A. halophilus* instead of *Aestuariibacter halophilus*.

14The effective publication states that the type strain was also deposited as IMSNU 14007, but no documentation was supplied.

15The effective publication states that the type strain was also deposited as NBRC 114670, but no documentation was supplied.

16The etymology should state L. gen. n. *piscis*, of a fish.

17The list editors corrected *Jeongeuplla* to *Jeongeupella* (Je.ong.eup.el’la. N.L. fem. n. *Jeongeupella*, …).

18The list editors corrected *suadae* to *suaedae* (su.ae’dae. N.L. gen. n. *suaedae*, of the plant genus *Suaeda*).

19In the protologue, the KCTC number is erroneously given as KCTC 92,072.

20The list editors corrected *flavus* to *flava* (fla’va. L. fem. adj. *flava*, yellow).

21The effective publication states that the type strain was also deposited as SDUM 810003, but no documentation was supplied.

22The list editors corrected *Planctoellipticum* to *Planctellipticum* (Planct.el.lip’ti.cum. … N.L. neut. n. *Planctellipticum* …).

23The preferred etymology is (ma.ior.i.cen’sis. M.L. fem. adj. *maioricensis*, …).

24The type of the genus was not explicitly stated in the article.

25In the protologue, the KCTC number is erroneously given as KCTC 43,249.

26In the abstract, the genus name is misspelled *Wenyingzhangia*.

The following correction should be made in Validation List 215 (https://doi.org/10https://doi.org/10.1099/ijsem.0.006173):

The type strains of *Entomospira entomophila* and *Entomospira nematocerorum* were deposited as NCCB 100892 and NCCB 100893, respectively.

The following corrections should be made in Validation List 216 (https://doi.org/10https://doi.org/10.1099/ijsem.0.006229):

The type strain of *Clostridium aromativorans* is WLY-B-L2 and not WKY-B-L2 as stated. The List Editors thank M. Sakamoto for pointing out the error.

*Onishia niordana* (Diéguez *et al*. 2020) de la Haba *et al*. 2024 must be corrected to *Onishia niordiana* (Diéguez *et al*. 2020) de la Haba *et al*. 2024 (basonym: *Halomonas niordiana* Diéguez *et al*. 2020).
